# Anti-angiogenic SPARC peptides inhibit progression of neuroblastoma tumors

**DOI:** 10.1186/1476-4598-9-138

**Published:** 2010-06-04

**Authors:** Alexandre Chlenski, Lisa J Guerrero, Radhika Peddinti, Jared A Spitz, Payton T Leonhardt, Qiwei Yang, Yufeng Tian, Helen R Salwen, Susan L Cohn

**Affiliations:** 1 Department of Pediatrics, University of Chicago, 900 East 57th Street, KCBD Rm. 5240, Chicago, IL 60637, USA; 2 Department of Pediatrics, Stroger Hospital, 1900 W Polk, Suite 1100, Chicago, IL 60612, USA; 3 Department of Pediatrics, University of Chicago, 900 East 57th Street, KCBD Rm. 5100, Chicago, IL 60637, USA

## Abstract

**Background:**

New, more effective strategies are needed to treat highly aggressive neuroblastoma. Our laboratory has previously shown that full-length Secreted Protein Acidic and Rich in Cysteine (SPARC) and a SPARC peptide corresponding to the follistatin domain of the protein (FS-E) potently block angiogenesis and inhibit the growth of neuroblastoma tumors in preclinical models. Peptide FS-E is structurally complex and difficult to produce, limiting its potential as a therapeutic in the clinic.

**Results:**

In this study, we synthesized two smaller and structurally more simple SPARC peptides, FSEN and FSEC, that respectively correspond to the N-and C-terminal loops of peptide FS-E. We show that both peptides FSEN and FSEC have anti-angiogenic activity *in vitro *and *in vivo*, although FSEC is more potent. Peptide FSEC also significantly inhibited the growth of neuroblastoma xenografts. Histologic examination demonstrated characteristic features of tumor angiogenesis with structurally abnormal, tortuous blood vessels in control neuroblastoma xenografts. In contrast, the blood vessels observed in tumors, treated with SPARC peptides, were thin walled and structurally more normal. Using a novel method to quantitatively assess blood vessel abnormality we demonstrated that both SPARC peptides induced changes in blood vessel architecture that are consistent with blood vessel normalization.

**Conclusion:**

Our results demonstrate that SPARC peptide FSEC has potent anti-angiogenic and anti-tumorigenic effects in neuroblastoma. Its simple structure and ease of production indicate that it may have clinical utility in the treatment of high-risk neuroblastoma and other types of pediatric and adult cancers, which depend on angiogenesis.

## Background

Neuroblastoma tumors exhibit a broad spectrum of clinical behavior, reflective of their biologic heterogeneity [1]. Although significant progress has been made in the successful treatment of neuroblastoma tumors with favorable biology, more effective therapeutic strategies are still needed for children with high-risk neuroblastoma [2]. A strong correlation between high-risk tumors and angiogenesis has been reported by us and other investigators [3,4], suggesting that blood vessels may be clinically relevant therapeutic targets. In support of this hypothesis, preclinical studies have demonstrated that neuroblastoma tumor growth can be significantly impaired following treatment with anti-angiogenic agents [5,6].

The histologic features of neuroblastoma tumors have also been shown to be prognostic, and an abundance of Schwannian stroma is associated with a more benign tumor phenotype and favorable prognosis [7]. Schwann cells are known to secrete factors that induce neuroblastoma differentiation [8,9]. Studies from our laboratory have demonstrated that Schwann cells also influence neuroblastoma tumor growth by secreting inhibitors of angiogenesis [9], the most potent of which is Secreted Protein Acidic and Rich in Cysteine (SPARC) [10]. SPARC belongs to a group of non-structural components of the extracellular matrix (ECM) that modulate interactions between cells and their environment [11,12]. It is highly expressed in a variety of cell types associated with remodeling tissues [13]. Although the mechanism for its anti-angiogenic activity is not well understood, SPARC is capable of interfering with the binding of angiogenic stimulators vascular endothelial growth factor (VEGF), platelet-derived growth factor (PDGF), and basic fibroblast growth factor (bFGF) to their receptors in endothelial cells, resulting in inhibited proliferation [14]. SPARC has also been shown to down-regulate VEGF in glioma cells [15].

The role of SPARC in tumorigenesis appears to be cell-type specific due to its diverse function in a given microenvironment [16]. In some types of cancer, high levels of SPARC expression have been shown to correlate with disease progression and poor prognosis. In melanoma cells, high levels of SPARC expression induce epithelial-mesenchymal transition and increases invasion and tumor progression [17,18]. High levels of SPARC are also associated with invasive meningioma [19] and osteosarcoma [20]. In glioma, SPARC promotes invasion, but delays tumor growth [21]. In other types of cancer, SPARC functions as a tumor suppressor. It inhibits the proliferation of breast cancer cells [22] and induces apoptosis in ovarian cancer cells [23]. In the majority of primary lung adenocarcinomas, SPARC silencing is associated with poor outcome [24]. In non-small cell lung cancer, SPARC expression is frequently down-regulated due to methylation of the tumor suppressor RASSF1A [25]. Similarly, in breast and prostate cancers and neuroblastoma, the majority of neoplastic cells do not express SPARC [12].

Previously, we synthesized peptides corresponding to the highly conserved structural domains of SPARC and tested their ability to inhibit angiogenesis [26]. To maintain the structural integrity of the native modules, cysteines within the peptides were linked with disulfide bonds during the synthesis. Minimal to no inhibitory activity was observed with the peptides corresponding to the Kazal module and the α-helix of the EC domain. In contrast, the epidermal growth factor (EGF)-like module peptide FS-E strongly inhibited endothelial cell migration *in vitro *and angiogenesis *in vivo*. Reduction of the two disulfide bonds in the FS-E peptide completely abrogated the angiogenesis inhibitory effects, indicating that structural conformation is critical for this biological activity. We have now designed two additional SPARC peptides that structurally correspond to N- and C-terminal loops of the FS-E peptide: FSEN and FSEC, respectively. These peptides are smaller, less structurally complex, and easier to produce than the FS-E peptide. We show that both peptides block angiogenesis, although FSEC is more potent. We also demonstrate that FSEC effectively inhibits neuroblastoma tumor growth in a preclinical model.

## Results

### Peptide design

**Figure 1:**
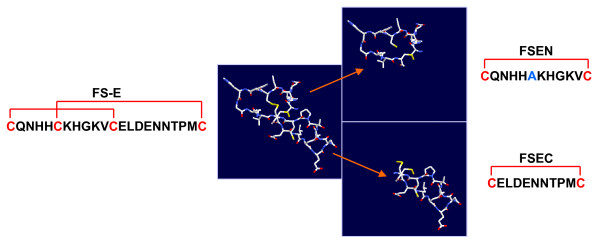
**SPARC peptides FSEN and FSEC**. SPARC peptides FSEN and FSEC were designed to correspond to the N-terminal and C-terminal loops of peptide FS-E, respectively. The native cysteine linkage was preserved in peptide FSEN; the unpaired cysteine was replaced with alanine. To maintain the conformation of peptide FSEC, cysteine 4 was linked with cysteine 3 instead of cysteine 2.

Our previous studies demonstrated that peptide FS-E, representing the N-terminal EGF-like module of the SPARC follistatin domain, potently inhibits angiogenesis [26]. This module of SPARC is a β-hairpin, highly twisted by disulfide bonds that link cysteine 1 to cysteine 3 and cysteine 2 to cysteine 4. The crystal structure of peptide FS-E shows that the two central cysteines are closely located (Figure 1). By linking cysteine 4 with cysteine 3 in lieu of cysteine 2, separate N- and C-terminal loops of the peptide can be produced without disturbing the native structure. Using this strategy, we synthesized the N- and C-terminal loops of peptide FS-E as two separate peptides, FSEN and FSEC, as detailed in Figure 1. Both peptides were folded into their native conformation by linking the cysteines that were placed at both ends. In the FSEN peptide, alanine was substituted for the unpaired cysteine. Corresponding scrambled control peptides, scFSEN and scFSEC that were designed to contain the same amino acids as peptides FSEN and FSEC in a random order, were synthesized without special modifications.

### Peptides FSEN and FSEC inhibit angiogenesis* in vitro* and *in vivo*

**Figure 2:**
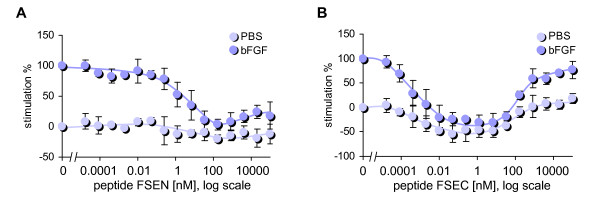
**SPARC peptides FSEN and FSEC inhibit endothelial cell migration**. HUVEC cells were treated with serial dilutions of SPARC peptides with or without 10 ng/ml bFGF in a modified Boyden chamber. Relative stimulation was calculated as percentage of bFGF-induced migration. (A) Peptide FSEN inhibited bFGF-stimulated endothelial migration with an EC_50 _of ~2 nM. (B) Peptide FSEC displayed a strong dose-dependent inhibition of bFGF-stimulated endothelial migration with an EC_50 _~1 pM. Dark circles represent bFGF-stimulated migration; light circles represent basal migration in the absence of an activator.

Migration assays were performed with human umbilical vein endothelial cells (HUVEC) and each of the synthetic SPARC peptides to characterize their anti-angiogenic properties *in vitro *and to compare their potencies with the original peptide FS-E. As shown in Figure 2, the C-terminal loop peptide FSEC inhibited bFGF-stimulated endothelial cell migration with an EC_50 _of ~1 pM, which was even lower than EC_50 _of ~10 pM of the original FS-E peptide. Higher concentrations of peptide FSEC did not inhibit endothelial cell migration, which is also a property of peptide FS-E [26] and the full-length SPARC [10]. Biphasic inhibition of endothelial cell migration is a characteristic feature of other natural inhibitors of angiogenesis, including thrombospondin-1 [27] and endostatin [28]. The N-terminal loop peptide, FSEN, showed weaker inhibition of bFGF-stimulated endothelial cell migration, with an EC_50 _of ~2 nM. Neither peptide, tested at concentrations from 0.1 pM to 100 μM, affected proliferation of HUVEC cells stimulated with bFGF (data not shown).

**Figure 3:**
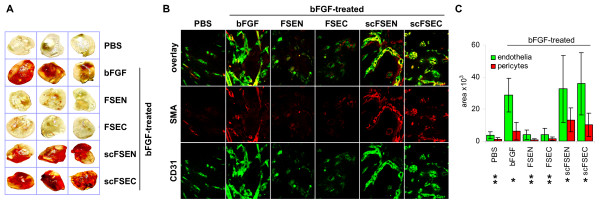
**Inhibition of neovascularization by peptides FSEN and FSEC in the Matrigel plug assay**. (A) Blood vessels developed for 7 days in nude mice, injected with Matrigel plugs containing 50 ng/ml bFGF alone (positive control), PBS (negative control), and bFGF with 10 μM SPARC peptides FSEN, FSEC, or scrambled control peptides scFSEN and scFSEC. (B) For quantitative analysis of angiogenesis and blood vessel architecture, endothelial cells were visualized with green CD31 immunofluorescence, and pericytes were detected with red SMA antibody. Representative photographs at ×400 magnification are shown. (C) The relative quantity of endothelial cells and pericytes was estimated by calculating the area occupied by green and red fluorescence (in pixels). There were statistically significant decreases in the blood vessel area and quantity of pericytes in the Matrigel plugs containing SPARC peptides compared to the positive control with bFGF alone (single asterisk) and from the negative control (double asterisk).

The anti-angiogenic properties of the peptides were further characterized *in vivo *using the Matrigel plug assay. Matrigel plugs containing bFGF and either SPARC peptides FSEN or FSEC were less hemorrhagic than the positive control Matrigel plugs containing bFGF with or without scrambled SPARC peptides (Figure 3A). To quantify the anti-angiogenic effects of the peptides, we stained the endothelial cells in paraffin sections of the Matrigel plugs with green fluorescence using anti-CD31 antibody and visualized the perivascular pericytes with red fluorescence using anti-α-smooth muscle actin (SMA) antibody (Figure 3B). The area occupied by each type of cells was quantified at low magnification in duplicate fields in each sample using ImagePro software. As shown in Figure 3C, the quantity of CD31-positive endothelial cells was statistically significantly decreased in the SPARC peptides-treated Matrigel plugs compared to the positive controls with bFGF alone (p < 0.005). Although the area, occupied by SMA-positive pericytes in the Matrigel plugs containing the SPARC peptides was significantly smaller compared to the positive controls (p < 0.005), there was no significant differences in the ratio of pericytes to endothelial cells (p > 0.8), indicating identical pericyte coverage. In the Matrigel plugs treated with scrambled peptides, no differences in endothelial cell (p > 0.7) or pericyte area (p > 0.3) were detected compared to the positive controls with bFGF alone.

### SPARC peptide FSEC potently inhibits neuroblastoma tumor growth

**Figure 4:**
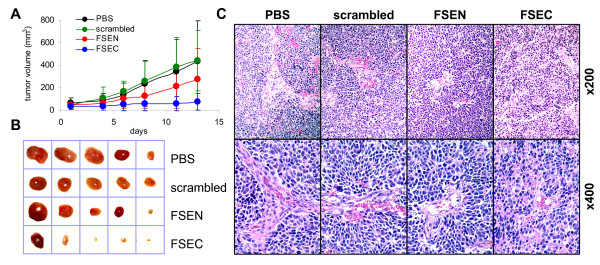
**Inhibition of neuroblastoma tumor progression by SPARC peptides FSEN and FSEC in the preclinical model of neuroblastoma**. (A) Mice with xenografted SMS-KCNR neuroblastoma cells received intraperitoneal injection of PBS, 10 mg/kg of the SPARC peptides FSEN or FSEC, or scrambled peptide scFSEN five times a week for 2 weeks. Treatment with the SPARC peptide FSEC resulted in a statistically significant (p < 0.05) decrease in the average size of tumors starting from day 4 until the end of the treatment period. The average tumor weight was reduced to 26% (p = 0.01) of average control tumor weight. The average weight of tumors treated with peptide FSEN was reduced to 88%, but the decrease was not statistically significant (p = 0.83). Scrambled peptide did not affect tumorigenicity of neuroblastoma xenografts (102%, p = 0.97). (B) Representative photographs of neuroblastoma tumors treated with the SPARC peptides. (C) Normalization of the blood vessels in the SPARC peptide-treated xenografts. H&E staining of areas with large blood vessels at ×200 and ×400 magnification shows areas of extensive hemorrhage and MVP in control tumors and tumors treated with the scrambled peptides. In contrast, no evidence of hemorrhage or MVP was seen the SPARC peptide-treated tumors.

To examine the anti-tumor activity of the SPARC peptides, mice with subcutaneous neuroblastoma xenografts were treated with either FSEN, FSEC, scrambled peptide scFSEN, or PBS 5 days/week for 2 weeks. Compared to control group, statistically significant inhibition of tumor growth was seen in the animals treated with the FSEC peptide, but not FSEN (Figure 4). In the FSEC-treated animals, the average tumor weight at the end of the experiment was 26% of the weight of the control tumors (0.38 ± 0.42 g vs 1.48 ± 1.24 g, respectively; p = 0.01). In the group of animals treated with the peptide FSEN, the average weight of tumors was 88% of the controls, which was not statistically significant (1.30 ± 1.77 g, p = 0.83). The average weight of tumors resected from animals treated with the scrambled peptide was not different from the PBS controls (1.51 ± 1 g, p = 0.97).

Vascular architecture was initially evaluated on H&E stained sections. In the control tumors, the blood vessels were structurally abnormal with dilated, tortuous appearance and prominent microvascular proliferation (MVP) (Figure 4C). In xenografts treated with scrambled peptide, a similar architecture was noted with disorganized layers of endothelial and perivascular cells. Multiple areas of hemorrhage were present in the control and scrambled peptide-treated tumors. In the SPARC peptides-treated tumors, the blood vessel architecture was more normal, and there was no evidence of hemorrhage or MVP.

**Figure 5:**
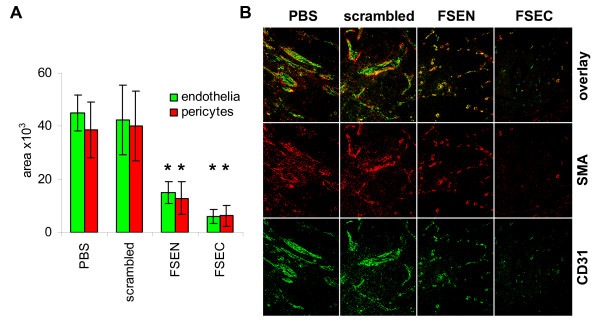
**Inhibition of tumor-induced angiogenesis by peptides FSEN and FSEC in the animal model**. For quantitative analysis of angiogenesis in the mouse xenografts, paraffin sections were stained with green CD31 and red SMA immunofluorescence. (A) Angiogenesis was quantified by calculating the area occupied by green CD31-positive endothelial cells and red SMA-positive pericytes. The quantity of tumor blood vessels was statistically significantly decreased in the SPARC peptide-treated xenografts compared to vehicle treated control (p < 0.001; marked with an asterisk). Treatment with the scrambled peptide did not affect angiogenesis in the xenografted tumors. (B) Representative photographs at ×100 magnification.

Immunofluorescent analysis of the tumors demonstrated a significant reduction in the quantity of endothelial cells in the SPARC peptide-treated xenografts compared to controls (Figure 5). The average area occupied by endothelial cells in FSEC-treated xenografts was 13 ± 6% of the blood vessel area in the control xenografts (5.9 ± 2.6 pixels ×10^3 ^vs 44.9 ± 6.6 pixels ×10^3^, respectively; p < 0.001). Peptide FSEN also inhibited angiogenesis in the xenografts, although less potently. The average area occupied by endothelial cells in the FSEN-treated tumors was 33 ± 9% of the control (15.0 ± 4.0 pixels ×10^3^; p < 0.001). In contrast, no significant difference in the quantity of endothelial cells in the xenografts treated with scrambled peptide or control vehicle was detected (42.4 ± 13.1 pixels ×10^3^, p = 0.67).

**Figure 6:**
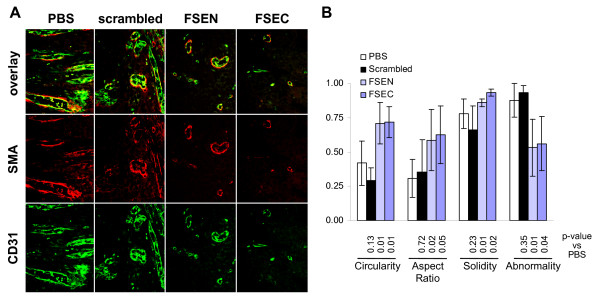
**Blood vessel architecture in the peptide-treated murine neuroblastoma xenografts**. (A) Endothelial cells and pericytes were visualized with green CD31 and red SMA staining respectively. Aberrant blood vessel architecture was evident at ×400 magnification in control xenografts treated with the vehicle or scrambled peptide. Peptide-treated tumors had more structurally normal, thin-walled blood vessels. (B) Quantitative analysis of blood vessel architecture. Circularity estimates roundness of an object, modified aspect ratio measures its elongation, and solidity approximates the density. On a scale from 0 to 1, where 1 is a perfect circle, all descriptors were significantly closer to 1 in the SPARC peptide-treated versus the control tumors, indicating that blood vessels are more round, less elongated and more compact. Vessel abnormality was calculated as 1 - (circularity × aspect ratio × solidity), and was significantly reduced in tumors treated with peptides FSEN and FSEC, showing that treatment induced normalization of tumor vasculature. All p-values versus PBS-treated control tumors are shown below the respective bars.

The quantity of perivascular cells in each group corresponded to the level of angiogenesis. The average area occupied by pericytes in the tumors treated with peptides FSEC vs control tumors was 16 ± 10% (6.2 ± 4.0 pixels ×10^3 ^vs 38.6 ± 10.4 pixels ×10^3^, respectively; p < 0.001) and 33 ± 16% (12.8 ± 6.2 pixels ×10^3^; p = 0.004) for peptide FSEN (Figure 5). No difference in the quantity of pericytes in tumors treated with the scrambled peptide and controls was detected (40.1 ± 13.1 pixels ×10^3^; p = 0.84). Although pericyte-to-endothelial cell ratio was not affected by the treatment, differences in the blood vessel architecture were apparent between the control and SPARC peptide-treated tumors. In the xenografts treated with vehicle or scrambled peptide, the blood vessels exhibited abnormal architecture with multiple layers of hypertrophic endothelial cells which often lacked co-localized pericytes. In contrast, blood vessels in the SPARC peptides-treated tumors had more normal architecture, mainly consisting of a single layer of spindle-shaped endothelial cells, enveloped by a layer of pericytes (Figure 6).

We used a novel approach to quantitatively assess blood vessel architecture by using 2-dimensional shape descriptors of Image J software that is similar to the method, previously described for 3-dimensional shapes [29]. Circularity is a measure of roundness of an object with value ranges from 0 to 1, where 1 is a perfect circle. As shown in Figure 6B, the blood vessels in the peptide-treated tumors are more circular compared to the PBS and scrambled peptide-treated controls (Figure 6B). Aspect ratio reflects the shape of an object as the ratio of its height to its width. We found that the aspect ratio was significantly closer to 1 in the treated versus the control tumors, indicating that these vessels are less elongated. Solidity, which approximates density of an object, was appreciably higher in the peptides-treated xenografts, compared to tumors treated with PBS or scrambled peptide, indicating that the endothelial cells are spaced more compactly in the blood vessels in the SPARC peptides-treated tumors. We integrated all three shape descriptors into a coefficient of abnormality to quantitatively assess roundness, elongation, and the border shape of the blood vessels using a scale from 0 to 1, with lower values reflective of more normal architecture. The scores for the blood vessels in the tumors treated with peptides FSEN and FSEC were significantly lower (0.53 ± 0.21 and 0.56 ± 0.20, respectively) compared to the vessels in tumors, treated with PBS or scrambled peptide (0.88 ± 0.12 and 0.93 ± 0.05, respectively).

## Discussion

We have previously shown that full-length SPARC and SPARC peptide FS-E, that corresponds to the highly conserved EGF-like module of the follistatin domain, potently inhibit angiogenesis and neuroblastoma tumor growth in preclinical models [10,26,30]. The structure of the FS-E peptide is complex, and we have demonstrated that its anti-angiogenic function is conformation-dependent [26]. In an effort to develop a therapeutic that may be suitable for clinical use, in this study we designed and synthesized two simply structured derivative peptides, FSEN and FSEC. Because proper structure is imperative to maintain activity of the FS-E peptide, peptides FSEN and FSEC were folded into their native conformation by linking the end cysteines during synthesis with disulfide bonds. This was possible due to the close proximity of the two central cysteines in the FS-E peptide [31]. Linking cysteine 4 with cysteine 3 instead of cysteine 2 allowed us to design peptides that correspond to amino acid sequences in the N- and C-terminal loops without disturbing the native folding. We found that both FSEN and FSEC function as inhibitors of angiogenesis, although the FSEC peptide was more potent.

Due to potent anti-angiogenic activity *in vitro*, a dose of 10 mg/kg was administered 5 days a week to investigate the effects of SPARC peptides on tumor progression in the preclinical model. This is a lower amount than the 10 to 100 mg/kg daily doses that have been used to test other anti-angiogenic peptides in preclinical studies [28,32-35]. At this low dose, peptide FSEC significantly suppressed neuroblastoma tumor growth in experimental animals. Furthermore, consistent with properties of SPARC as an inhibitor of angiogenesis, the number of endothelial and perivascular cells was also significantly decreased in the peptide-treated animals, compared to controls.

Although the anti-tumor and anti-angiogenic effects of peptide FSEN were less potent, both SPARC peptides had profound effects on the architecture of tumor-induced blood vessels. In contrast to the structurally abnormal blood vessels that were seen in the control tumors, thin walled blood vessels were detected in the peptide-treated tumors, suggesting that treatment with FSEN and FSEC induced blood vessel normalization. Interestingly, hemorrhage was not detected in the peptide-treated tumors, whereas significant hemorrhage was detected in the control tumors.

Similar to the original SPARC peptide FS-E [26], the proper structural conformation may be essential to maintain the anti-angiogenic activity of the FSEC and FSEN peptides. Disulfide bonds are likely to be unstable in the reducing environment *in vivo*, leading to poor pharmacokinetic properties and decreased activity of the peptides. Stable analogs of disulfide bonds have been used to increase activity of other biologically active peptides [36], and we plan to use this approach to produce more stable analogs of peptides FSEC and FSEN.

It is well established that cancer blood vessels are not structurally normal [37]. In tumors, multiple layers of hypertrophic endothelial cells alternate with areas in which endothelial cell coverage is lacking. Corresponding abnormalities in the deposition of the basement membrane are also commonly observed. Perivascular smooth muscle cells, which provide both mechanical and physiological support for the endothelial monolayer in normal blood vessels, fail to co-localize with endothelial cells in neoplastic blood vessels. These abnormalities disrupt the integrity of the blood vessels, resulting in a heterogeneous blood supply of the tumor tissue, vessel leakiness, and hemorrhage. Our recent evaluation of blood vessel architecture in a series of neuroblastoma tumors demonstrated that structurally abnormal blood vessels are commonly seen in high-risk tumors, and the presence of MVP was statistically significantly associated with decreased survival [38].

Normalization of neoplastic blood vessels has been demonstrated with other anti-angiogenic therapeutics [37], and recently the extent of vascular normalization following treatment with an anti-VEGF therapy was shown to be predictive of outcome in patients with glioblastoma [39]. Emerging evidence also indicates that by normalizing the abnormal structure and function of tumor vasculature, anti-angiogenic agents can alleviate hypoxia and increase the efficacy of conventional therapies [37]. A recently completed phase I dose-escalation study of an anti-VEGF agent has provided evidence of both vascular normalization and sensitization of rectal tumors to radiation [40,41]. Using a simple method to quantitatively assess blood vessel architecture, we show that treatment with either FSEC or FSEN similarly normalizes the tumor vasculature. Normalization of blood vessels may enhance drug delivery to the tumor tissue and improve efficacy of chemotherapeutic agents. A better understanding of the molecular and cellular basis of vascular normalization may ultimately lead to more effective strategies for combining chemotherapy and radiation with agents that are capable of inducing blood vessel normalization.

## Conclusion

In summary, we have designed and synthesized SPARC-derived peptides FSEN and FSEC that are easy to produce and demonstrated that they function as inhibitors of angiogenesis *in vitro *and *in vivo*. The potent anti-tumor activity of SPARC peptide FSEC in a preclinical model of neuroblastoma indicates that it may be an effective treatment for children with high-risk neuroblastoma, as well as other malignancies. Similar to other anti-angiogenic agents, the SPARC peptides induced normalization of blood vessels and thus may enhance drug delivery to the tumor tissue. Preclinical studies testing the activity of peptide FSEC in combination with chemotherapy are planned.

## Materials and methods

### Peptide synthesis

SPARC peptides FSEN (CQNHHAKHGKVC) and FSEC (CELDENNTPMC) were synthesized at Alpha Diagnostics International (San Antonio, TX) using fmoc/tboc chemistry as cyclic cysteine-linked molecules, with disulfide bonds unambiguously formed during peptide synthesis. The purity of the peptides was assessed by high-performance liquid chromatography, and the molecular mass was checked by mass spectrometry. Control scrambled peptides scFSEN (KCGHKHQCAVHN) and scFSEC (MEPECNLNCTD), which contain the same amino acids as peptides FSEN and FSEC in a random order, were made without special modifications.

### Cell lines

HUVEC cells (VEC Technologies, Rensselaer, NY) were maintained at 5% CO_2 _in EGM media (Lonza, Walkersville, MD) supplemented with 5% FBS (Life Technologies, Carlsbad, CA). Neuroblastoma cell line SMS-KCNR was grown at 5% CO_2 _in RPMI 1640 (Life Technologies) supplemented with 10% heat-inactivated FBS and 1% penicillin/streptomycin.

### Endothelial cell migration assay

To characterize the anti-angiogenic properties of the peptides *in vitro*, migration assays were performed in a modified Boyden chamber with HUVEC cells in EBM media (Lonza) containing 0.01% BSA as described [42]. Serial dilutions of each of the synthetic SPARC peptides, starting at 100 μM were assayed with or without 10 ng/ml bFGF (National Cancer Institute Preclinical Repository, Frederick, MD). At least three independent experiments were performed for each peptide. To compare multiple experiments, the data were represented as the percentage of bFGF-induced stimulation using the difference between bFGF-induced migration and background migration in EBM alone as 100% control.

### Endothelial cell proliferation assay

The effect of SPARC peptides on endothelial cell proliferation was measured in 96-well plates with HUVEC cells in EBM media supplemented with 0.5% FBS. Serial ×10 dilutions of SPARC peptides from 0.1 pM to 100 μM with and without 10 ng/ml bFGF were added to quadruplicate wells, cells were grown at 37°C in 5% CO_2 _for 24 and 48 h, and proliferation was determined with the CellTiter cell proliferation assay (Promega, Madison, WI).

### Matrigel assay

To characterize the anti-angiogenic properties of the peptides *in vivo*, Matrigel assays were performed in 4- to 6-week-old homozygous athymic nude mice (Harlan, Madison, WI). SPARC peptides FSEN, FSEC and corresponding scrambled peptides scFSEN and scFSEC were added to 0.4 ml of Growth Factor-reduced Matrigel (BD Biosciences, Bedford, MA) containing 10 units/ml heparin and 50 ng/ml bFGF to a final concentration of 10 μM. At least 5 animals were subcutaneously injected with Matrigel plugs containing bFGF and each SPARC peptide. Matrigel plugs with bFGF and no peptides served as positive controls. Matrigels without either bFGF or peptides were used as negative controls. Mice were sacrificed 7 days after the Matrigel injections, gels were recovered by dissection, photographed, fixed in formaldehyde, and embedded in paraffin.

### Xenograft model

A neuroblastoma xenograft model was used to examine the anti-tumor activity of peptides FSEN and FSEC. Briefly, 4-6 week old athymic nude mice were injected subcutaneously with 0.2 ml PBS containing 1 × 10^7 ^SMS-KCNR neuroblastoma cells. Once tumors were palpable (~70 mm^3^), animals were randomized into four treatment groups with at least 6 animals per group. Experimental animals received intraperitoneal injection of 10 mg/kg of the SPARC peptides FSEN or FSEC, or scrambled peptide scFSEN (0.3 mg of the peptide in 150 μl of PBS) 5 days a week for 2 weeks. Animals in the control group were injected with PBS without the peptide. Possible toxic effects of the treatment were assessed by monitoring the animals for signs of distress such as difficulty in ambulating, ulceration, cachexia, respiratory distress, loss of 15-20% body weight, or gain of 10% body weight. The size of the tumors was determined every 2-3 days by external measurements with a caliper. At the end of treatment, animals were euthanized using CO_2 _followed by cervical dislocation. Tumors were removed, measured, weighed, and photographed. Tissue was fixed with 10% buffered formalin, embedded in paraffin and 4 μm-thick sections were prepared for histologic evaluation. All animal studies were approved by the IACUC at the University of Chicago.

### Histological analysis and immunofluorescence

Four-μm-thick sections were stained with H&E for histological evaluation. The entire tissue section was evaluated for vascular morphology. Vessels with thickened walls containing complete layers of hypertrophied endothelial cells plus additional layers of vascular mural cells were classified as positive for MVP as previously described [38]. Vessels with normal vessel architecture and no more than a single layer of flat, spindle shaped endothelial cells were characterized as MVP negative. For quantitative analysis of angiogenesis, 4-μm-thick sections were stained with anti-CD31 antibody (Santa Cruz Biotechnology Inc., Santa Cruz, CA) at a 1:50 dilution followed by FITC-labeled secondary antibody. To characterize the blood vessel architecture, pericytes were visualized with red fluorescence using anti-α-SMA antibody (Sigma-Aldrich, St. Louis, MO) at 1:100 dilution. The area occupied by each cell type was quantified at ×100 magnification in duplicate fields in each sample using ImagePro software. Two-dimensional shape descriptors of Image J software were used to quantitatively assess blood vessel architecture. Circularity was calculated as 4π × area/perimeter^2^, modified aspect ratio was measured as object's width/length, and solidity was calculated as a ratio of an object area/convex hull (outermost outline). Coefficient of abnormality was calculated as 1 - (circularity × aspect ratio × solidity).

### Statistical analysis

All *in vitro *experiments were repeated at least in triplicates and standard deviations were calculated. All animal studies had at least 5 mice per group and mean values of the tumor volumes, weights, and vessel densities were compared. All the quantitative values obtained in the experiments were evaluated using paired Student's t-test. A p-value of 0.05 was required to ascertain statistical significance.

## Competing interests

A provisional patent application has been filed for the SPARC peptides FSEN and FSEC. The authors declare no additional conflicting interests in relation to the described work.

## Authors' contributions

SC conceived and supervised the study, AC and LJG designed the peptides, performed *in vitro *experiments, imaging studies and data analysis, RP performed pathological analysis, JAS, PL and HRS assisted with *in vitro *studies, QY and YT did the xenograft studies. All authors participated in preparation of the manuscript.
